# Gold Nanoparticle Delivery of Modified CpG Stimulates Macrophages and Inhibits Tumor Growth for Enhanced Immunotherapy

**DOI:** 10.1371/journal.pone.0063550

**Published:** 2013-05-15

**Authors:** Adam Yuh Lin, Joao Paulo Mattos Almeida, Adham Bear, Nathan Liu, Laureen Luo, Aaron Edward Foster, Rebekah Anna Drezek

**Affiliations:** 1 Department of Bioengineering, Rice University, Houston, Texas, United States of America; 2 Center for Cell and Gene Therapy, Baylor College of Medicine, Houston, Texas, United States of America; University of Queensland, Australia

## Abstract

Gold nanoparticle accumulation in immune cells has commonly been viewed as a side effect for cancer therapeutic delivery; however, this phenomenon can be utilized for developing gold nanoparticle mediated immunotherapy. Here, we conjugated a modified CpG oligodeoxynucleotide immune stimulant to gold nanoparticles using a simple and scalable self-assembled monolayer scheme that enhanced the functionality of CpG *in vitro* and *in vivo*. Nanoparticles can attenuate systemic side effects by enhancing CpG delivery passively to innate effector cells. The use of a triethylene glycol (TEG) spacer on top of the traditional poly-thymidine spacer increased CpG macrophage stimulatory effects without sacrificing DNA content on the nanoparticle, which directly correlates to particle uptake. In addition, the immune effects of modified CpG-AuNPs were altered by the core particle size, with smaller 15 nm AuNPs generating maximum immune response. These TEG modified CpG-AuNP complexes induced macrophage and dendritic cell tumor infiltration, significantly inhibited tumor growth, and promoted survival in mice when compared to treatments with free CpG.

## Introduction

Synthetic oligodeoxynucleotides (ODNs) containing the unmethylated cytosine-phosphate-guanine (CpG) motif are potent stimulants of the innate immune system. These sequences bind to Toll-like receptor 9 (TLR9) in the endosome of antigen presenting cells (APCs), thus promoting the expression of co-stimulatory molecules, the secretion of inflammatory cytokines, and the development of CD8^+^ T cell responses [1], [2]. As a result, CpG ODNs have shown great promise as a monotherapy and as a vaccine adjuvant for the treatment of cancer [3]–[5]. Although many studies have focused on the effects that CpG ODNs have on B cells and plasmacytoid dendritic cells (pDCs), these sequences also have important effects on macrophages and myeloid derived suppressor cells (MDSCs). For instance, the antitumor effects of CpG immunotherapy in weakly immunogenic tumors are mainly mediated by macrophages as opposed to T cells [6]. CpG ODNs can directly inhibit the immunosuppressive functions of MDSCs and cause them to differentiate into macrophages with antitumor activity [7]. CpG ODNs can also suppress MDSC activity by indirectly stimulating pDCs to produce interferon-α (IFNα) which in turn promotes MDSC differentiation [8]. Thus, targeting these immune cells in the tumor microenvironment is clinically relevant.

As nanoparticles are naturally cleared by macrophages, dendritic cells (DCs), and other APCs [9]–[11], they are excellent carriers for CpG delivery to innate immune cells. Liposomal nanoparticle encapsulation methods enhanced the immune stimulatory effect of CpG and promoted antitumor activity when combined with ovalbumin immunization [12]. Bourqin and colleagues also demonstrated that ovalbumin immunization combined with CpG encapsulating gelatin nanoparticles produced significantly higher activation of CD8^+^ T cells than when combined with free CpG oligos [13]. In addition, the encapsulation of CpG reduced the systemic release of pro-inflammatory cytokines and attenuated systemic side effects such as lymphoid follicle destruction and splenomegaly [13]. Similarly, Kwong *et al*. found that CpG and anti-CD40 monoclonal antibody encapsulated in liposomes were more effective than free CpG and induced significantly lower levels of IL-6 and TNFα in the serum [14]. Currently, however, the use of nanocarrier delivered CpG has not been tested as a monotherapy against cancer. Also, these encapsulation methods generate particles ranging from 100–200 nm in diameter, far from the optimal 50–60 nm size range for maximum particle uptake [15].

Gold nanoparticles (AuNPs) can be easily functionalized with thiol-modified synthetic oligonucleotides to form a self-assembled monolayer [16], making them useful platforms for the delivery of CpG ODN. AuNPs are also desirable vehicles because they are inert, biocompatible, and possess optical properties tunable for diagnostic and photothermal applications [17], In addition, DNA strands that are conjugated on AuNPs are more resistant to nuclease degradation [18]. Most importantly, AuNPs are readily taken up by immune cells [11], [19] and collect in endosomes [15], [20], [21], thereby facilitating access to TLR9 within antigen presenting cells. Given these characteristics, we hypothesize that CpG-coated AuNPs can enhance delivery of CpG to the target TLR9 receptor, thus enhancing the therapeutic effect of the oligonucleotide.

We developed a modified CpG ODN conjugated gold nanoparticle design to target innate immune cells *in vitro* and *in vivo* in order to mount an anti-tumor immune response. The design is optimized to maintain DNA content on the particle and to promote cellular uptake. We show that CpG conjugated AuNPs significantly enhance macrophage stimulation *in vitro* and inhibit tumor growth *in vivo* when compared to treatments with the equivalent dose of free CpG. The antitumor effect of the CpG-AuNP particles is potent and does not require combination treatment, suggesting that these complexes are clinically applicable and can be used for CpG monotherapy.

## Materials and Methods

### Cell Culture

The macrophage cell line J774.A1 (ATCC) was maintained in Dulbecco’s Modified Eagle Medium (DMEM), supplemented with 10% Fetal Bovine Serum (FBS) and 1% penicillin/streptomycin. The B16-OVA cell line was kindly provided by Dr. Xiao-Tong Song (Baylor College of Medicine) [22] and cultured in Dulbecco’s Modified Eagle Medium (DMEM), supplemented with 10% FBS, 2 mM Glutamax (Invitrogen, Carlsbad, CA), and 0.5 mg/ml Geneticin (Invitrogen). The cells were maintained at 37°C and 5% CO_2_.

### Particle Synthesis

Citrate stabilized gold nanoparticles (15 nm, 30 nm, and 80 nm) were purchased from Ted Pella. Modified CpG 1826 designs were purchased from Integrated DNA Technology (IDT). All reagents were purchased from Sigma Aldrich unless specified otherwise. All DNA types were uncapped by incubation with 100 mM dithiothreitol in sodium phosphate solution, pH 8.5, and eluted though illustra NAP-5 columns (GE Healthcare) with sodium phosphate solution, pH 6.5, after 1 hr incubation at 25°C. Uncapped CpG sequences (0.5 µM end concentration) were added to citrate stabilized gold nanoparticles for 24 hrs. The solution was brought to 1x phosphate buffered saline (PBS) and 0.1% Tween 20 and placed on a nutator for another 24 hrs. The particles were then collected and washed with PBS through three centrifugation steps. 15 nm particles were spun at 13,200 g for 20 min, 30 nm particles were spun at 7,000 g for 20 min, and 80 nm particles were spun at 1,000 g for 20 min.

### CpG 1826 Sequences

Three different designs were conjugated on gold nanoparticles. Design 1: 5′-HS-C6-TCCATGACGTTCCTGACGTT-3′. Design 2: 5′-HS-C6-TTTTTTTTTTT-TCCATGACGTTCCTGACGTT-3′. Design 3 (tmCpG): 5′-HS-C6-TTTTTTTTTTT-(CH_2_CH_2_O)_3_-TCCATGACGTTCCTGACGTT-3′.

### CpG Content on AuNP Measurements

The particle concentration of 15 nm or 30 nm AuNPs conjugated with CpG of varying designs were calculated by comparing the optical density of the solution with that of the purchased AuNP stock solution. The particles were then incubated with 1.4 mM mercaptoethanol for 48 hours. After incubation, the particles were spun at 16,000 g for 10 minutes. Using the absorbance of the supernatants at 260 nm and the extinction coefficients of each DNA, as provided by IDT, we calculated the concentration of DNA in the supernatants. The CpG concentration and gold nanoparticle concentration ratio gave the number of DNA per AuNP.

### Stimulation with CpG ODN and AuNP CpG Particles

J774.A1 macrophage cells were seeded at 1×10^5^ cells/ml in 12 well plates and cultured for 2 days. The cells were then exposed to their respective treatment conditions in triplicate and incubated for 24 hours. After incubation, the cell supernatants were collected and stored at –80°C prior to analysis. The concentration of nanoparticles added was standardized by total surface area to deliver the same dose of CpG. For 15 nm particles, 4×10^11^ particles/ml were used, for 30 nm 10^11^ particles/ml were used, and for 80 nm 1.4×10^10^ particles/ml were used.

### Cytokine Concentration Measurement

The supernatants were analyzed for TNFα using an enzyme linked immunosorbent assay (ELISA) kit (R&D Systems), following the manufacturer’s instructions. IL-6 and G-CSF were analyzed using a 32-plex murine cytokine/chemokine array (Millipore).

### Mice and Tumor Model

C57BL/6J mice (Jackson Laboratories, Bar Harbor, ME) were maintained in the pathogen-free mouse facility at Rice University. This study was approved by the Institutional Animal Care and Use Committees (IACUC) of Rice University (#A12041201). B16-OVA tumors were formed in the flank of mice through subcutaneous injection of 5×10^5^ cells. The length and width of tumors were subsequently measured 3 to 4 times a week using a digital caliper. Once the tumors reached approximately 15 mm^2^ in size, the CpG treatments were applied. The mice received either intratumor injections of PBS (PBS condition), 6.4 µg CpG 1826 (Free CpG condition), or 10^13^ tmCpG-AuNP particles (tmCpG-AuNP condition). The doses were repeated on days 4 and 7 after the first dose. Mice were sacrificed once the area of the tumor reached 1 cm^2^, per IACUC requirements. CpG sequences used in *in vivo* applications had phosphorothioate modifications to minimize degradation.

### Tumor Immune Infiltration Analysis

As with the tumor growth study, mice were implanted with 5×10^5^ B16-OVA cells in the flank. The mice received 3 injections of PBS (n = 4), free CpG (n = 5), free tmCpG (n = 4), or tmCpG-AuNP (n = 5) once the tumors reached an area of 15 mm^2^. After 24 hours, the mice were euthanized and the tumors were harvested and passed through 70 µm cell strainers (BD Falcon). The cells were stained with antibodies against CD8, CD4, CD11b, CD11c, and Gr-1 (BD Biosciences) and analyzed using a BD FACSCanto II flow cytometer.

### Statistics

All statistical analyses were done using JMP Pro Software. Significance was assigned at the α = 0.05 level. The comparisons between the cytokine secretions caused by the different designs and nanoparticle sizes were done using Tukey’s HSD test. Comparisons among the conditions inducing immune cell infiltration were also done using Tukey’s HSD test. A student’s t test was done to calculate the differences in tumor growth. Differences in survival were assessed using the Log Rank test.

## Results

### Rationale for CpG Conjugated AuNP Designs

**Figure 1:**
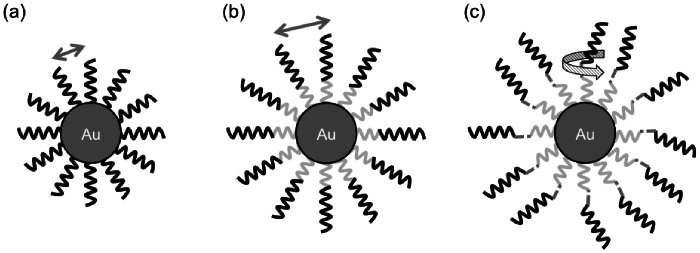
CpG AuNP conjugate design schematics. (a) Design 1, CpG-SH, directly has CpG (black lines) assembled on the AuNP surface. (b) Design 2, CpG-T11-SH, incorporates a poly-T nucleotide spacer (light gray lines) to increase the spacing between CpGs (straight arrows). (c) Design 3, CpG-TEG-T11-SH, adds a triethylene glycol (dark gray dotted line) between the CpG sequence and the nucleotide spacer to allow rotation of the CpG segment (curved arrow).

DNA coated gold nanoparticles have been heavily studied and often utilize self-assembling properties of the natural formation of thiol-gold dative bonds [18], [23], [24]. These studies show that modification of the functional DNA on the gold nanoparticles can maximize its function. Therefore, we examine three different CpG designs to extrapolate the optimal construct (Figure 1). In the following sections, a common CpG (1826) was used (5′-TCCATGACGTTCCTGACGTT-3′) for ease of comparison.

The first design is the most simple of the three. It incorporates a thiol group modification on the 5′ end of the CpG sequence (CpG-SH), allowing CpGs to form a self-assembled monolayer directly on the AuNP surface. The main disadvantage of this design is that the space between the CpGs may be too small for efficient TLR9 binding. Therefore, to improve the spacing between the DNAs, 11 thymidine (poly-T) nucleotides are inserted between the thiol modification and the CpG sequence (CpG-T11-SH). This second design has been used and optimized in several DNA-DNA or DNA-RNA binding constructs for detection or silencing. However, for the case of CpGs, we are examining DNA-receptor binding interactions. The rigidity of the DNA strands may hinder the binding of the CpGs to TLR9s. Thus, for the third design, a triethylene glycol (TEG) spacer is included between the poly-T and the CpG sequence (CpG-TEG-T11-SH) with the expectation that the TEG modification would allow free rotation and further improve binding to TLR9.

### Characterization of CpG Conjugated AuNP Designs

Prior to assessing the functional efficacy of the designs, the stability and DNA content of the three CpG conjugated AuNP constructs were evaluated using three different AuNP sizes: 15 nm, 30 nm, and 80 nm in diameter. For the different core sizes, salting the particles during the assembling process is important for a successful DNA coating [25]. Since AuNPs greater than 20 nm benefit from raising the salt concentration gradually [26], the salt concentration of the 30 nm and 80 nm CpG conjugated AuNP constructs were increased slowly over one and a half hour, while the 15 nm constructs were salted all at once.

The stability of the particle constructs were compared to the citrate stabilized nanoparticles by analyzing the absorbance spectra (Figure S1). The spectra of the DNA coated AuNP constructs red shifted 4–5 nm for all core sizes except for the CpG-80 nm AuNP. The CpG-80 nm AuNP aggregated and no peak was detected. Therefore, the CpG-80 nm AuNPs were excluded from further experiments. The shifts, however, suggests successful conjugation of the DNA onto AuNPs. There was no broadening of the peaks, which suggests no aggregation of these particles.

**Figure 2:**
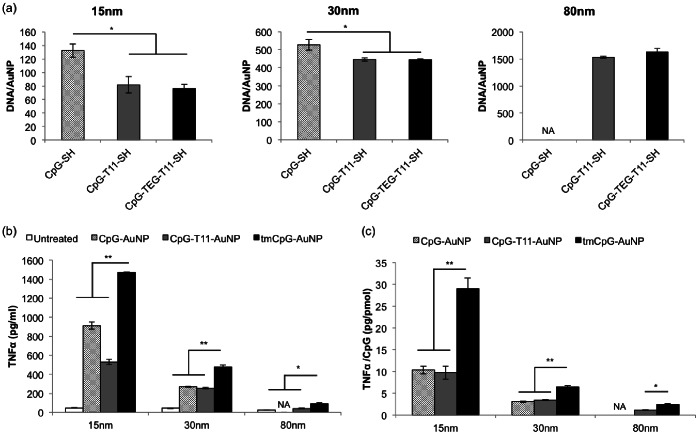
DNA content characterization and functional TNFα assays of CpG AuNP conjugated constructs. (a) CpG AuNP conjugate designs and DNA content of each CpG design on the 15 nm, 30 nm and 80 nm AuNPs (*p<0.05). 80 nm AuNP CpG-SH construct aggregated and thus the DNA content was not measured. (b) TNFα levels from macrophage stimulation by CpG conjugated AuNP designs for the three particle sizes (*p<0.05; **p<0.01). (c) TNFα levels normalized to amount of CpG presented by the nanoparticles (*p<0.05; **p<0.01).

Furthermore, to determine the amount of DNA conjugated on AuNPs of each design, the CpG strands were removed from the particle surface through place exchange by mercaptoethanol. The concentration of DNA in the solution was calculated by measuring the absorbance values at 260 nm after removal of the particles (Figure 2A). There was no significant difference of CpG content per AuNP between the CpG-T11-SH design and the design containing the TEG modification (CpG-TEG-T11-SH) for all core sizes. This shows that the TEG modification did not alter the DNA assembling process on the gold nanoparticles. Conversely, the design with CpG-SH (132 DNA/15 nm-AuNP and 528 DNA/30 nm-AuNP) showed significantly higher loading on AuNPs compared to the other two designs for both 15 nm and 30 nm core AuNPs (p = 0.02; p = 0.04). The CpG-T11-SH design contained 82 DNA/15 nm-AuNP and 447 DNA/30 nm-AuNP, and the CpG-TEG-T11-SH design contained 76 DNA/AuNP and 445 DNA/30 nm-AuNP. These numbers are consistent with previous reports by Demers *et al*. describing the surface density of DNA on AuNPs with and without nucleotide spacers [25], [27].

### Functional Evaluation of CpG-AuNPs Designs in vitro

The efficacies of the different designs were evaluated by their ability to stimulate murine macrophages to secrete tumor necrosis factor-α (TNFα) *in vitro*. TNFα is a cytokine secreted by activated macrophages and is an important component of the anti-tumor activity of macrophages [6]. The CpG-conjugated nanoparticles for all constructs were incubated with the macrophages overnight and the TNFα levels in the media supernatant were measured using enzyme-linked immunosorbent assays (ELISA). The particles concentration was standardized by overall surface area in order to normalize the total amount of CpG delivered to the macrophages. The CpG-TEG-T11-AuNP design, or TEG modified CpG AuNPs, (hereby referred to as tmCpG-AuNPs) showed significantly higher macrophage stimulation compared to the other two designs at almost 1,400 pg/ml for 15 nm constructs (p<0.0001), 480 pg/ml for 30 nm constructs (p<0.0001), and 90 pg/ml for 80 nm constructs (p = 0.004) (Figure 2c). From these results, we can conclude that the tmCpG design is the most effective construct independent of core size.

It is interesting that the CpG-AuNP design (800 pg/ml) caused higher stimulation than the CpG-T11-AuNP design (420 pg/ml) for 15 nm constructs (p<0.0001). A similar trend was noticed by Wei and colleagues between the CpG-T11-AuNP and CpG-AuNP designs [28]. However, 30 nm constructs did not share the same trend (p = 0.84). This outcome can be explained by the previous DNA content results (Figure 2a). For the 15 nm constructs, the CpG-AuNP design had more DNA per particle than the CpG-T11-AuNP design. Therefore, when comparing the amount (pg) of TNFα secreted per pmol of CpG delivered, one finds no significant functional difference between the CpG-AuNP and CpG-T11-AuNP designs, which also holds true for 30 nm constructs. The tmCpG-AuNPs, however, caused significantly higher stimulation and TNFα secretion, approximately three times higher, compared to the other two designs (p<0.0005), again indicating that the particles containing the TEG modification were the most effective and displayed the highest functionality (Figure 2c). In addition to confirming that the tmCpG was the most effective design, the TNFα results demonstrated that tmCpG-15 nm AuNPs were significantly better than the 30 nm and 80 nm constructs (Figure 2B/C). However, since the experiments prior were done separately, a combined TNFα stimulatory experiment was performed using 15 nm, 30 nm and 80 nm tmCpG-AuNP constructs. To ensure that the 15 nm core size supremacy was not specific to TNFα, multiplex ELISAs were used to investigate the core size effects on other cytokines. Furthermore, to ensure that the stimulatory effect is specific and not caused just by the presence of DNA on the particles, control CpG sequences were conjugated on gold nanoparticles and used for stimulation experiments. These sequences were identical except that the cytosine and guanine bases were in reverse order (5′-TCCATGAGCTTCCTGAGCTT-3′).

**Figure 3:**
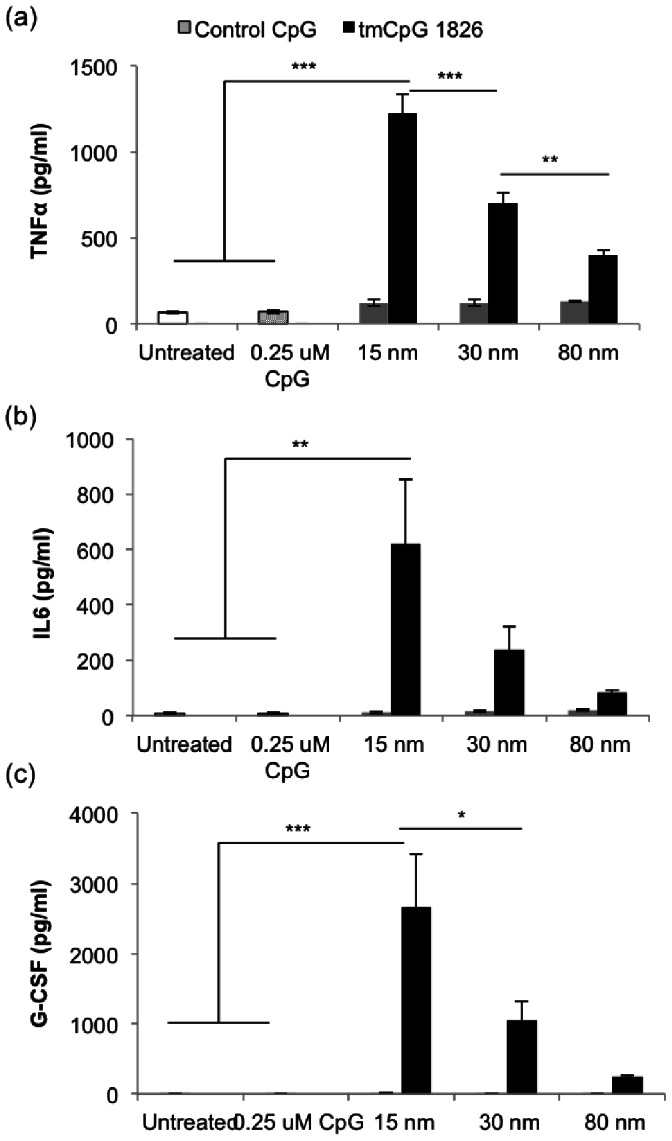
Cytokine and growth factor secretion following stimulation with free CpG, AuNPs coated with control tmCpG, or AuNPs coated with tmCpG for 15 nm, 30 nm and 80 nm core sizes. (a) TNFα secretion, (b) IL-6 secretion, and (c) G-CSF secretion (pg/ml) (***p<0.0001; **p<0.01; *p<0.05).

The tmCpG-AuNPs of all sizes still cause significantly higher TNFα release than the equivalent concentration of free CpG (Figure 3a). This concentration of free CpG is the end concentration of CpG expected based on the total amount of CpG added to the particles during the conjugation process. Finally, the 15 nm particles proved to be the optimal size for CpG delivery, demonstrating significantly higher stimulation than 30 nm and 80 nm particles. A similar effect was observed when measuring the concentration of IL-6, an inflammatory cytokine known to be up-regulated in macrophages following CpG stimulation [29], [30] (Figure 3b). AuNP delivery also promoted the expression of granulocyte-colony stimulating factor (G-CSF), a growth factor that promotes hematopoietic progenitor cell circulation and that has been shown to be up-regulated by CpG *in vivo* [31] (Figure 3c). The control sequence nanoparticles, conversely, did not cause a significant increase in TNFα secretion compared to untreated controls, demonstrating that there was not non-specific stimulation caused by DNA on AuNPs.

Additionally, we assessed the effect of the three CpG sequence designs in solution, without being conjugated to the nanoparticles, to ensure that modifications themselves did not induce macrophage stimulation. None of the modifications on the CpG sequences caused TNFα secretion. Finally, we incubated citrate particles of all three sizes with the macrophages and found that the particles alone did not induce TNFα secretion (Figure S2). Given these *in vitro* results, the 15 nm tmCpG-AuNP design was chosen for the following *in vivo* experiments.

### AuNP-CpG Inhibits Tumor Growth and Promotes Survival in mice Bearing B16-OVA tumors

**Figure 4:**
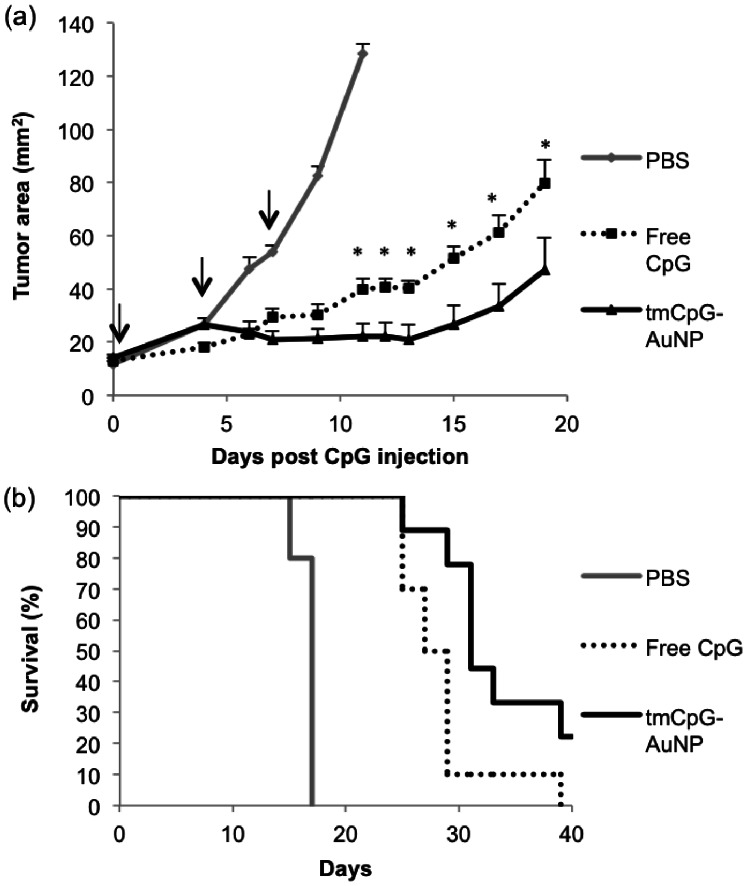
*In vivo* anti-tumor effect following intratumor injections on days 0, 4, and 7, as indicated (arrows), with PBS, free CpG or tmCpG- AuNP. (a) Tumor growth and (b) survival percentage after the first CpG injection (*p<0.05).

C57BL/6 mice were implanted with 5×10^5^ B16-ovalbumin (B16-OVA) tumor cells subcutaneously. Once the tumors reached a size of ∼15 mm^2^, we injected approximately 10^13^ particles into the tumor, equivalent to a dose of about 6.4 µg CpG. We administered the CpG treatment via intratumor injections because this route has been shown to be superior to intravenous or subcutaneous injections [32], [33]. Subsequent doses were injected on days 4 and 7 after the first dose, as was done in a previous intratumor study [33]. Mice receiving equivalent doses of free CpG or receiving injections of PBS were used as controls. Starting on day 11 after the first dose (day 17 overall), the tmCpG-AuNP treatment induced significant inhibition of tumor growth when compared to free CpG (p = 0.0306) (Figure 4a). Both the free CpG treatment and the tmCpG-AuNP treatment induced significant tumor inhibition when compared to the PBS treatment (p<0.0001). The difference between free CpG treatment and AuNP treatment remained significant throughout the study (p = 0.043 on day 19 after first injection). We also found that a single intratumor injection of 30 nm tmCpG-AuNP significantly inhibited tumor growth when compared to PBS treated mice (p = 0.0124) (Figure S3).

In the control group (n = 5), the first PBS treated mouse reached the pre-defined 1 cm^2^ tumor limit on day 15 of the study, and the remainder reached the limit on day 17 (Figure 4b). In the free CpG group (n = 10), the percentage of mice under the limit dropped to 70% on day 25. The remaining mice reached the tumor limit by day 39. In contrast, the percentage of mice under the limit in the AuNP condition (n = 9) remained higher than the free CpG condition throughout the study. Two mice (22%) showed no measurable tumor growth after treatment and remained under the limit until the end of the study on day 47. Overall, the AuNP treatment promoted significantly higher survival than the free CpG treatment (p = 0.0164).

### CpG Treatment Induces Immune Cell Infiltration of the Tumor

To elucidate the immune mechanism of tumor growth inhibition, we analyzed the infiltration of immune cells at the tumor site using flow cytometry. Mice were again implanted with B16-OVA tumors and received the same 3 dose treatment regimen of PBS (n = 4), free CpG (n = 5), free tmCpG, or tmCpG-AuNP (n = 5) once the tumors reached a size of approximately 15 mm^2^. As was done *in vitro*, the free tmCpG condition was included to ensure that the modifications on the CpG sequence were not the cause of any immune response. The tumors were harvested 24 hours after the last treatment injection, and the cells were then re-suspended and stained for CD8 (cytotoxic T cells), CD4 (helper T cells), CD11c (dendritic cells), and CD11b (macrophage) expression, as well as CD11b and Gr-1 co-expression (myeloid derived suppressor cells).

**Figure 5:**
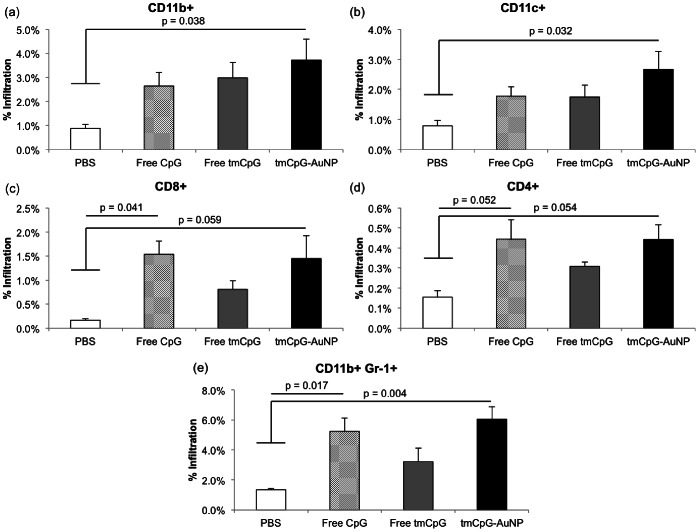
Percent tumor infiltration immune cells. (a) CD11b^+^ cells, (b) CD11c^+^, (c) CD8^+^ cells, (d) CD4^+^ cells, and (e) CD11b^+^Gr-1^+^ cells.

Although tmCpG-AuNP treatment shows no significant difference in immune cell infiltration when compared to free CpG, it shows significantly higher infiltration of CD11b^+^ (p = 0.0377), CD11c^+^ (p = 0.0323), and CD11b^+^/Gr-1^+^ cells when compared to the PBS treated condition (Figure 5). The free CpG condition showed significantly higher infiltration of CD8^+^ T cells (p = 0.0414) and CD11b^+^/Gr-1^+^ cells (p = 0.0168) when compared to the PBS control. The free tmCpG showed no significant infiltration of any immune cells when compared to the PBS treated mice.

## Discussion

The immune response following CpG treatment has been characterized in a variety of tumor models, and a number of immune populations have been implicated in antitumor activity. Treatment of murine colon adenocarcinoma was shown to be mainly mediated by CD8^+^ T cells with partial effect from innate effector cells [34], while the antitumor response against large B16 melanoma tumors was dominated by macrophages [6]. Most recently, it has been shown that CpG treatment can also inhibit the suppressive activity of myeloid derived suppressor cells (MDSCs), a population that inhibits T cell activity [7], [8]. However, effective CpG treatment in mice commonly requires high doses given repeatedly, raising concerns of possible systemic toxicity [35]. Consequently, a number of studies have focused on the use of nanoparticles to promote delivery of CpG to APCs, thereby enhancing its stimulatory effect. The nanoparticle formulations explored include gelatin nanoparticles [13], liposomes [14], [36], DNA origami structures [37], and most recently, gold nanoparticles [38], but these were only explored in the context of combination treatments.

The stronger anti-tumor response of CpG bound to gold nanoparticles compared to free oligonucleotides illustrates the utility of gold nanoparticles for delivery of CpG and, potentially, other immune stimulatory agents. Lee and colleagues assessed gold nanoparticle delivery of red fluorescent protein and CpG on the same particle *in vivo* and found encouraging results. However, in their study the addition of CpG on the antigen AuNPs showed only a modest improvement of the anti-tumor response at a single time point. Furthermore, adding CpG on the particle had no effect compared to antigen only particles when the mice were immunized and then challenged with tumor cells [38]. Finally, the effectiveness of the CpG-AuNP complexes alone was not evaluated. We posit that the therapeutic efficacy of CpG-AuNP particles can be improved and optimized through simple design alterations. Here, we assessed CpG-AuNP complexes’ effectiveness by comparing its anti-tumor effects to equivalent doses of free CpG. We demonstrated that the oligonucleotide structure and particle size can be designed so as to make CpG-AuNP complexes effective for monotherapy. The design considerations discussed here show that gold nanoparticles can be optimized for immune stimulant delivery.

For DNA-receptor interactions, spacing between the CpG strands can be crucial for maximum efficacy, especially since TLR9 is an endosome membrane receptor. Using a nucleotide spacer reduces “steric crowding” of the DNA strands, thus making the target sequence more accessible to binding [25], [27]. Nucleotide spacers have been used widely to improve functionality of DNA on AuNPs for various applications such as antisense gene modulation [18]. The choice of nucleotide spacer is important; poly-adenine (poly-A) spacers yield lower nanoparticle surface coverage than poly-thymidine (poly-T) spacers. This effect is due to adenine’s stronger affinity to gold, which causes poly-A spacers to lie down on the particle and restrict oligonucleotide access to the surface [27]. Efforts to further improve binding efficacy come at a cost of reducing the amount of DNA on the particle. Rosi *et al*. show that using tetrathiol-modified DNA with nucleotide spacers further improves the functionality of antisense oligonucleotides while sacrificing the number of strands delivered per particle by roughly 50% [18]. Wei and colleagues used a T_20_/A_20_ duplex spacer linked to CpGs to improve its functionality *in vitro* when compared to a T_20_ spacer [28]; however, the use of double strand (0.11 DNA/nm^2^) as opposed to a single strand DNA (0.19 DNA/nm^2^) reduces the surface density on gold surfaces [39] and reduces the uptake of nanoparticles by approximately 50% [40]. Giljohann *et al*. noted that higher DNA densities on AuNPs caused increased cellular uptake [41]. Therefore, designing a CpG modified AuNP to improve CpG functionality of the poly-T modified CpG without sacrificing the amount of DNA per particle is critical for maximum efficacy. We incorporated a short triethylene glycol (TEG) spacer in between the poly-T and the CpG sequence in the tmCpG design to address that issue. This design proved to be the most effective, generating the highest secretion of cytokines per pmol of CpG delivered. The large difference between efficacies of the poly-T spacer alone CpG design versus the incorporation of TEG could be caused by the increased rotation and mobility of the extended CpG strand. Having free moving CpGs can improve binding to TLR9, which is confined in the endosomal membrane.

As mentioned above, particle size can dramatically affect particle uptake and thus affect CpG delivery and functional efficacy. The macrophage stimulatory effect of tmCpG-AuNPs is improved by using 15 nm particles, likely because these particles are more easily taken up [28], [42]. In addition, smaller particles have greater curvature and thus provide more space for binding between the DNA strands. Overall, the use of gold nanoparticles for immune modulation is clinically valuable not only because of the enhanced therapeutic effects, but also because of the facile synthesis and tuning of the complex. AuNPs can be easily functionalized and tuned to the desired size, making our design reproducible and scalable.

The optimal 15 nm TEG modified design proved to be effective *in vivo*, significantly inhibiting tumor growth and promoting survival when compared to free CpG. Tuning of the oligonucleotide sequence and particle size permits the successful application of CpG-AuNP complexes as a monotherapy. The ability to enhance therapeutic activity through simple design alterations shows that gold nanoparticles are powerful carriers and that the design of AuNP-oligonucleotide complexes needs to be carefully considered for optimal therapeutic effect. In this study the significant anti-tumor activity observed in the free CpG condition when compared to PBS controls was unexpected given the low dose of CpG applied. However, the presence of the foreign ovalbumin antigen is likely to promote a strong response compared to what has been observed in other cancer models. Nevertheless, our results indicate that conjugation to AuNP enhances the anti-tumor effect of intratumor CpG injections. We also observed that the free CpG treated tumors were visually similar in shape and height (spherical) compared to the untreated tumors, while the tmCpG-AuNP treated tumors lacked structure or were flattened.

The tmCpG-AuNP treatment caused significantly higher infiltration of macrophages (CD11b^+^ cells) and dendritic cells (CD11c^+^) when compared to PBS treated mice. The treatment showed a trend towards higher infiltration of CD8^+^ T cells as well, but the finding was not significant (p = 0.0591). Interestingly, the tmCpG-AuNP and free CpG condition caused significant infiltration of CD11b^+^/Gr-1^+^ myeloid derived suppressor cells (MDSCs), an immune suppressive population that is known to promote tumor growth. However, as aforementioned, CpG treatment has been shown to reduce the suppressive activity of MDSCs. Therefore, even though the inflammatory response induced by CpG may attract infiltration of MDSCs, the treatment may be inhibiting the immune suppressive activity of these cells. For instance, Zoglmeier et al. report that CpG treatment does not reduce the percentage of splenic MDSCs in tumor bearing mice but does inhibit their ability to suppress T cell proliferation [8]. Future studies isolating tumor and splenic MDSCs following free CpG and tmCpG-AuNP injections can elucidate whether the treatments used here can inhibit MDSC activity.

Overall, the anti-tumor activity of the tmCpG-AuNP treatment appears to be mediated by the significant infiltration of macrophages and dendritic cells to the tumor site. We did not observe significant differences in infiltration between the free CpG and the tmCpG-AuNP conditions and thus cannot ascertain the immunological differences that may have made the tmCpG-AuNP treatment more effective *in vivo.* The increased efficacy of the tmCpG-AuNP may result from its effect on immune suppressive populations such as MDSCs or regulatory T cells; characterizing whether AuNPs can enhance the inhibitory effect that CpG has on MDSC activity merits further work.

In conclusion, CpG oligonucleotides are immune stimulatory agents that have shown clinical promise as single treatments and as vaccine adjuvants [4], [5]. However, CpG treatment can be limited by the need for high doses and by non-specific toxicity [35], such as systemic cytokine increase and coagulation inhibition [5]. Nanotechnology can address such concerns by enhancing delivery of CpG to antigen presenting cells, and a number of nanocarriers have been explored for this purpose [13], [14], [36]–[38]. Here we show that gold nanoparticles are an effective CpG carrier, enhancing the effect of CpG treatment both *in vitro* and *in vivo.* We developed a new design utilizing a poly-T and TEG spacer that enhances CpG functionality without lowering DNA content on the gold nanoparticle. In addition, we demonstrate that a monotherapy of AuNP-delivered CpG can inhibit tumor growth and promote survival when compared to the equivalent dose of free CpG. Future studies will explore AuNP delivered CpG in combination treatments and in metastatic disease models.

### Supporting Information Available

The supporting information includes absorbance data on the particles, *in vivo* data for 30 nm tmCpG-AuNP particles, and control experiments on modified CpG sequences and citrate particles.

## Supporting Information

Figure S1 **Absorbance spectra of CpG conjugated gold nanoparticle constructs (15** **nm, 30** **nm and 80** **nm) before and after assembly.**
(TIF)

Figure S2 **TNFα levels in macrophages following incubation with modified CpG sequences and citrate particles of 15 nm, 30 nm, and 80 nm diameters.**
(TIF)

Figure S3 **In vivo anti-tumor effect following a single intratumor injection of 30** nm tmCpG-AuNPs compared to free CpG and PBS (*p = 0.0124).
(TIF)
