# Supplementary figures and images for: Gold Nanoparticle Delivery of Modified CpG Stimulates Macrophages and Inhibits Tumor Growth for Enhanced Immunotherapy
Source: PLoS One. 2013 May 15;8(5):e63550. doi: 10.1371/journal.pone.0063550 (PMC3655178; doi:10.1371/journal.pone.0063550)

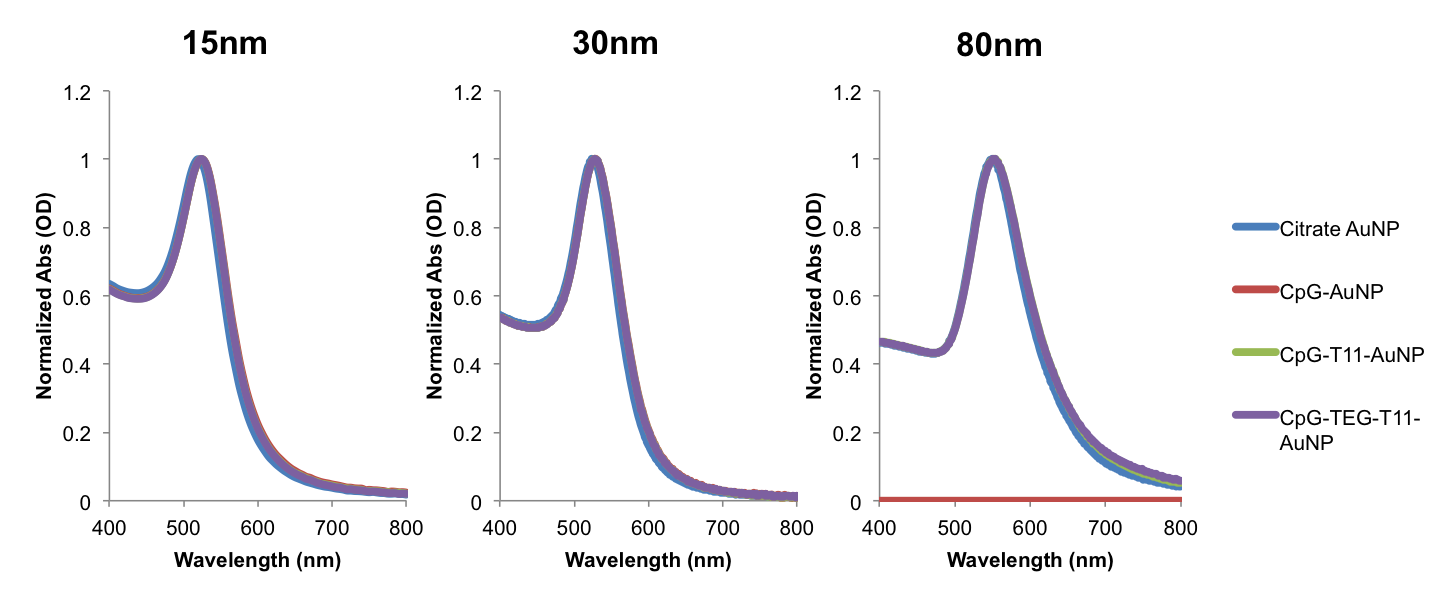

Supplement: Figure S1 — Absorbance spectra of CpG conjugated gold nanoparticle constructs (15 nm, 30 nm and 80 nm) before and after assembly. (TIF) [file pone.0063550.s001.tif]
